# Impact of lung function impairment after allogeneic hematopoietic stem cell transplantation

**DOI:** 10.1038/s41598-022-18553-6

**Published:** 2022-08-19

**Authors:** Yuya Kishida, Naoki Shingai, Konan Hara, Makiko Yomota, Chika Kato, Satoshi Sakai, Yasuhiro Kambara, Yuya Atsuta, Ryosuke Konuma, Atsushi Wada, Daisuke Murakami, Shiori Nakashima, Yusuke Uchibori, Daishi Onai, Atsushi Hamamura, Akihiko Nishijima, Takashi Toya, Hiroaki Shimizu, Yuho Najima, Takeshi Kobayashi, Hisashi Sakamaki, Kazuteru Ohashi, Noriko Doki

**Affiliations:** 1 Hematology Division, Tokyo Metropolitan Cancer and Infectious Diseases Center, Komagome Hospital, 3-18-22 Honkomagome, Bunkyo-ku, Tokyo, 1138677 Japan; 2 Department of Economics, University of Arizona, Tucson, AZ USA; 3 Division of Respiratory, Tokyo Metropolitan Cancer and Infectious Diseases Center, Komagome Hospital, Bunkyo-ku, Tokyo, Japan

**Keywords:** Outcomes research, Haematological cancer

## Abstract

Late-onset noninfectious pulmonary complications (LONIPC) are a major cause of morbidity and mortality after allogeneic hematopoietic stem cell transplantation (HSCT). However, the clinical impact of lung function deterioration itself in long-term adult survivors of HSCT remains to be fully investigated. This retrospective, longitudinal study aimed to investigate pulmonary function following HSCT in terms of its change and the clinical significance of its decline. We examined 167 patients who survived for at least 2 years without relapse. The median follow-up period was 10.3 years. A linear mixed-effects model showed that the slope of pulmonary function tests values, including percent vital capacity (%VC), percent forced expiratory volume in one second (%FEV_1_), and FEV_1_/forced VC ratio (FEV_1_%), decreased over time. The cumulative incidence of newly obstructive and restrictive lung function impairment (LFI) at 10 years was 15.7% and 19.5%, respectively. Restrictive LFI was a significant, independent risk factor for overall survival (hazard ratio 7.11, *P* = 0.007) and non-relapse mortality (hazard ratio 12.19, *P* = 0.003). Our data demonstrated that lung function declined over time after HSCT and that the decline itself had a significant impact on survival regardless of LONIPC.

## Introduction

Allogeneic hematopoietic stem cell transplantation (HSCT) is a curative treatment for hematological disorders. Advances in HSCT procedures, such as a less toxic conditioning regimen, anti-infection prophylaxis, and graft-versus-host-diseases (GVHD) management, have increased the number of long-term HSCT survivors^1,2^. If the original disease does not relapse within 2 years after HSCT, 85% of recipients survive for more than 10 years^3^. However, pulmonary complications are frequently encountered in HSCT and remain a major cause of morbidity and mortality^4,5^. Late-onset noninfectious pulmonary complications (LONIPC) are especially associated with a significantly poorer prognosis, larger healthcare resource utilization, and increased financial burden^6^.

Pre-transplant pulmonary dysfunction is linked to development of LONIPC as well as poorer outcomes^7,8^. Thus, pulmonary function tests (PFT) are performed before HSCT to predict pulmonary complications. Post-transplant PFT has also been shown to be predictive of LONIPC, especially bronchiolitis obliterans (BO)/bronchiolitis obliterans syndrome (BOS)^9–11^. However, because LONIPC rarely occurs beyond 2 years after HSCT^12,13^, the observational periods of previous studies were relatively short. Moreover, data on long-term pulmonary function transition in HSCT recipients are scarce. Although pulmonary function declines in survivors of childhood, adolescent, and early adulthood HSCT^14–16^, it is undetermined in older patients. Furthermore, the clinical significance of post-transplant pulmonary dysfunction itself in long-term survivors remains unknown. Therefore, this study aimed to investigate pulmonary function following HSCT in terms of its transition and the clinical significance of its decline.

## Patients and methods

### Patients

Patients who underwent their first HSCT at Tokyo Metropolitan Cancer and Infectious Diseases Center Komagome Hospital between July 2004 and July 2012 and survived relapse-free for at least 2 years after HSCT were included^3,17^. The exclusion criteria were death from any cause, relapse of the underlying disease or requirement of a second HSCT within 2 years of the first HSCT, followed up < 2 years after HSCT, or unavailability of pre- or post-HSCT PFT data. Transplantation procedures have been described in detail elsewhere^18,19^.

This study was approved by the Institutional Ethics Committee of Tokyo Metropolitan Cancer and Infectious Diseases Center Komagome Hospital (approval number 2651) and performed in accordance with the principles of the Declaration of Helsinki.

### Pulmonary function tests and pulmonary complications

Pulmonary function was assessed using a multi-functional spirometer (CHESTAC-8800 and CHESTAC-8900; CHEST MI, Inc., Tokyo, Japan) following the guidelines of the Japanese Respiratory Society, which agree with those of the American Thoracic Society and the European Respiratory Society^20,21^. A PFT was routinely performed 1 month before HSCT and again after HSCT as part of long-term follow-up (LTFU), in accordance with the guideline recommendations^22^. To account for the effects of aging, vital capacity (VC) and forced expiratory volume in one second (FEV_1_) were evaluated and expressed as a percentage of the predicted, normal values after matching age, height, and weight to those of healthy individuals. In addition to percent VC (%VC) and percent FEV_1_% (%FEV_1_), FEV_1_/forced VC ratio (FEV_1_%) was also assessed. A slight decline in pulmonary function was considered to have no clinical impact, especially if the pulmonary function remained within the normal range. Therefore, the clinical impact of lung function impairment (LFI) was analyzed using the standard values^23,24^; restrictive LFI was defined as %VC < 80% and obstructive LFI was defined as FEV_1_% < 70%.

BO/BOS was diagnosed based on clinical presentation, while the PFT, and CT findings were interpreted as per the National Institutes of Health consensus^25,26^. Although histopathological confirmation was not always performed, biopsy-proven BO was included as BO/BOS regardless of the PFT value. Interstitial lung disease (ILD) and organizing pneumonia (OP) were diagnosed using standard criteria^27^. All LONIPCs, other than BO/BOS were either ILD or OP. Because of the few cases of each event, we treated these events as ILD/OP in the analysis.

### Statistical analysis

Linear mixed-effects (LME) model-fitting using the maximum likelihood *t* test and Satterthwaite’s method, was employed to assess the correlation between repeated measures (change in %VC, %FEV_1_, and FEV_1_% from transplantation to the last follow-up) and years after HSCT. The LME model accounts for fixed and random effects and is used especially in regression analysis involving dependent data from multiple observations in each subject^28^. Thus, this method is effective for investigating PFT values after HSCT because the baseline characteristics and interval between HSCT and PFT differed from patient to patient.

The cumulative incidence of a post-HSCT event (BO/BOS, ILD/OP, obstructive LFI, restrictive LFI, and relapse) was estimated using Gray’s method, considering death and a second HSCT without an event as a competing event^29^. Patients who had obstructive or restrictive LFI before HSCT were excluded from each analysis. Since there is a pathophysiological correlation between BO/BOS and obstructive LFI, patients with BO/BOS were excluded from causative factor analysis for obstructive LFI. For the same reason, patients with ILD/OP were also excluded from analysis for restrictive LFI. The cumulative incidence of BO/BOS or ILD/OP was estimated in all patients regardless of pre-transplant lung function. Non-relapse mortality (NRM) was defined as death without disease relapse or progression. The Fine–Gray model was used to estimate the hazard risk of these events^29^. Overall survival (OS) was defined either as the time from the date of the first HSCT until death from any cause and censored on the date the patient was last known to be alive. Data from patients who received a second HSCT were censored on the date of the second HSCT. All events were initiated by the date of HSCT. The final date of observation was April 30, 2021. For survival analysis (OS and NRM), patients who had any LFI before HSCT were excluded. Cox proportional hazards regression analysis was used to examine the relationship between the factors and OS. The results are as a hazard ratio (HR) with a 95% confidence interval (95% CI). The covariates in the univariate and multivariate models included: age (≥ 50 years), sex (female vs. male), refined disease risk index (low/intermediate vs. high/very high)^30^, hematopoietic cell transplantation comorbidity index (0–2 vs. ≥ 3)^31^, human leukocyte antigen (HLA) compatibility, donor type, stem cell source, conditioning regimen (myeloablative vs. non-myeloablative)^32^, total body irradiation (TBI) in conditioning, smoking history (pack-years), acute GVHD, chronic GVHD, BO/BOS, ILD/OP, restrictive LFI, and obstructive LFI. Post-HSCT events (acute GVHD, chronic GVHD, BO/BOS, ILD/OP, restrictive LFI, and obstructive LFI) were treated as time-dependent covariates.

All outcome analyses were performed on the date of HSCT. Statistical significance was set at *P* < 0.05. Statistical analyses were performed on EZR, a graphical user interface for R, version 4.1.2 (http://www.r-project.org)^33^.

## Results

In total, 201 patients received their first HSCT and survived for at least 2 years without relapse, need for a second HSCT or loss to follow-up. Five patients were excluded owing to unavailability of pre-transplant PFT data and 29 due to unavailability of post-transplant PFT data, leaving 167 eligible patients. Table 1 summarizes the patient and transplant characteristics. The median age at transplant was 40 years (range: 15–71 years), and the median follow-up post-HSCT was 10.3 years (range: 2.2–16.2 years). Eleven patients reported relapse of the underlying disease, and nine of them received a second HSCT. A myeloablative conditioning regimen was employed for 84% of the patients. The most frequent indication for HSCT was acute myeloid leukemia (n = 43; 34%), and the most frequent stem cell source was bone marrow (n = 121; 72%).

**Table 1:** Patients characteristics.

Factors	Median value (range)/number (%)
Age at transplantation	41 (15–71)
**Sex**	
Female	63 (38)
Male	104 (62)
**Disease**	
Aplastic anemia	14 (8)
Acute lymphoblastic leukemia/lymphoma	43 (26)
Acute myeloid leukemia	57 (34)
Chronic myeloid leukemia	18 (11)
Myelodysplastic syndromes	30 (18)
Myeloproliferative neoplasms	3 (2)
Non-Hodgkin lymphoma	2 (1)
**Refined disease risk index**	
Low/intermediate	140 (84)
High/very high	27 (16)
**HCT-CI**	
< 3	153 (92)
≥ 3	14 (8)
**Pulmonary function test before HSCT**	
%VC	113.2 (63.4–155.3)
FEV_1_%	109.5 (49.1–85.4)
%FEV_1_	108 (49.2–153.6)
**HLA compatibility**	
Mismatch	56 (34)
Match	111 (66)
**Donor type**	
Related	47 (28)
Unrelated	120 (72)
**Stem cell source**	
Bone marrow	121 (72)
Peripheral blood	29 (17)
Cord blood	17 (10)
**Conditioning regimen**	
Myeloablative	141 (84)
Non-myeloablative	26 (16)
**TBI in conditioning**	
No	69 (41)
Yes	98 (59)

*HCT-CI* Hematopoietic Cell Transplantation Comorbidity Index, *HSCT* hematopoietic stem cell transplantation, *VC* vital capacity, *FEV*_*1*_ forced expiratory volume in 1 s, *HLA* human leukocyte antigen, *TBI* total body irradiation.

A total of 34 LONIPCs were diagnosed in 29 patients. Diagnoses included 17 cases of BO/BOS, 9 of ILD, 3 of OP, 3 of pleuroparenchymal fibroelastosis (PPFE), and 2 cases of air leak syndromes (ALS). PPFE and ALS were observed in patients with ILD. Histological confirmation was performed for seven patients (one with BO, four with ILD, and two with OP).

### Changes in pulmonary function tests

**Figure 1:**
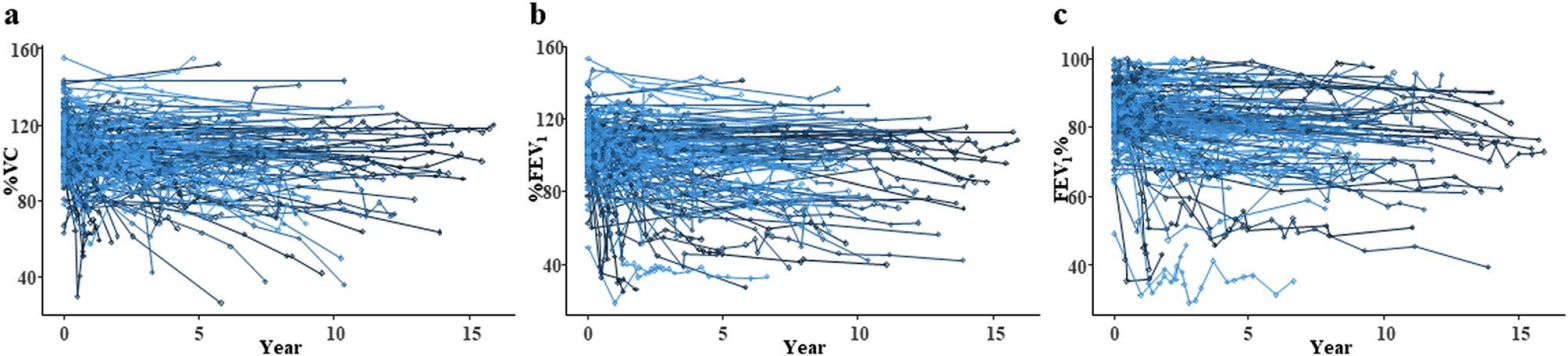
Transition of pulmonary function test values (**a**) percent vital capacity (%VC), (**b**) percent of predicted forced expiratory volume in one second (%FEV_1_), and (**c**) FEV_1_/forced VC ratio (FEV_1_%).

In total, 791 PFTs were performed over the study period. The median, minimum, and maximum number of PFTs per patient after HSCT was 4, 2, and 20, respectively. A total of 29 (17.4%) patients received 2 PFTs. Among these cases, PFT was performed at a median of 3.2 years (range: 0.2–15.7 years). The last PFT was performed at a median of 6.8 years (range: 0.2–15.9 years) post-HSCT. The transition of PFT values is shown in Fig. 1 [(a) %VC, (b) %FEV_1_, and (c) FEV_1_%]. During the study period, %VC decreased by > 10% in 58 patients and > 20% in 33 patients. Moreover, %FEV_1_, decreased by > 10% in 28 patients and > 20% in 12 patients. FEV_1_% decreased by > 10% in 63 patients and > 20% in 38 patients. Table 2 shows the results of the LME model for %VC, %FEV_1_, and FEV_1_%. %VC gradually declined after HSCT (mean slope difference: − 1.16; 95% CI: − 1.63 to − 0.70; *P* < 0.001). %FEV_1_ (mean slope difference: − 1.18; 95% CI: − 1.64 to − 0.74; *P* < 0.001) and FEV_1_% (mean slope difference: − 0.54; 95% CI: − 0.76 to − 0.32; *P* < 0.001) also declined.

**Table 2:** Linear mixed model analyses for pulmonary function after HSCT.

Factors	Estimated value (95% CI)	*P* value
**Mean slope difference per year**		
%VC	− 1.16 (− 1.63 to − 0.70)	< 0.001
%FEV_1_	− 1.18 (− 1.64 to − 0.74)	< 0.001
FEV_1_%	− 0.54 (− 0.76 to − 0.32)	< 0.001

*HSCT* hematopoietic stem cell transplantation, *CI* confidence interval, *VC* vital capacity, *FEV*_*1*_ forced expiratory volume in one second.

### Lung function impairment

A total of 41 patients developed LFI. Among them, 21 (51.2%) patients did not develop LONIPC during the long follow-up periods. Among the 20 patients who developed both LFI and LONIPC, the interval between these two events was within 1 month in 10 (50.0%) patients. There was more than a 1-year interval in 6 (30.0%) patients.

**Figure 2:**
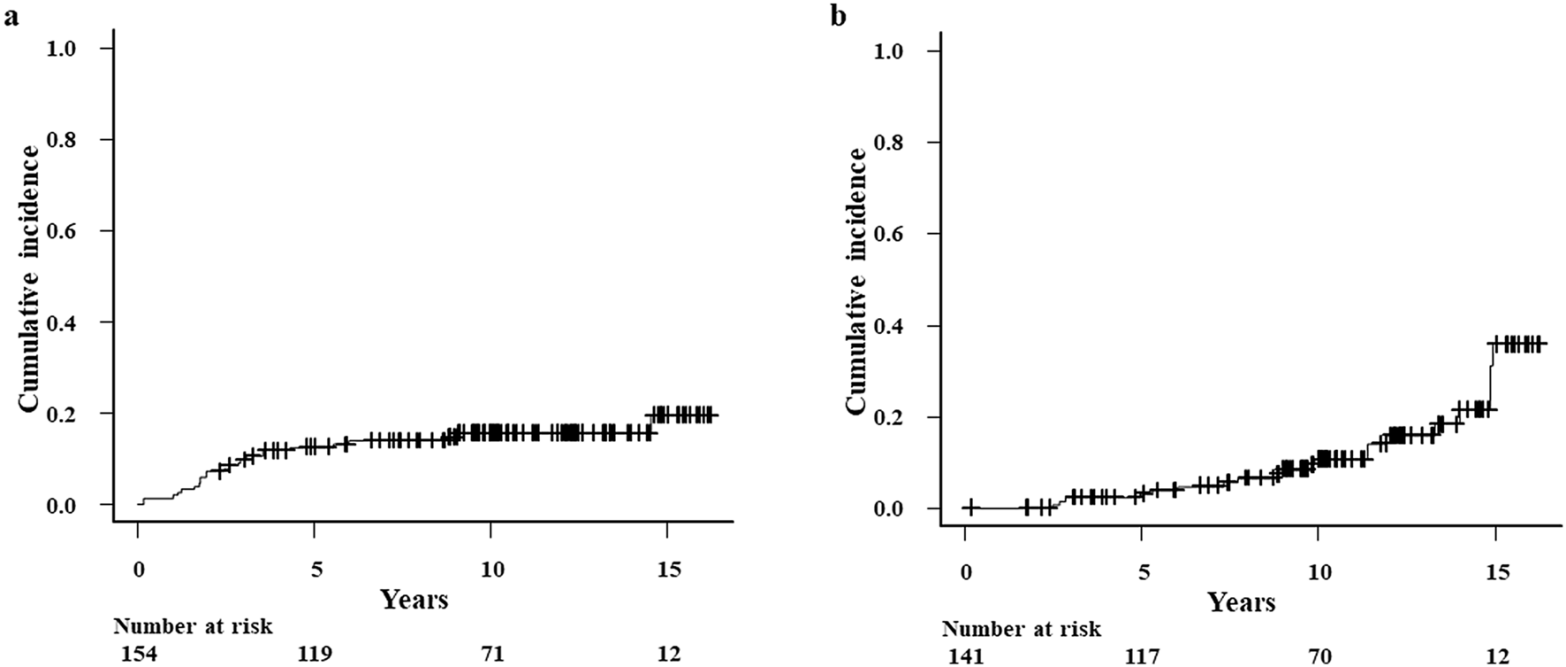
Cumulative incidence of obstructive lung function impairment. (**a**) Patients including those with bronchiolitis obliterans/bronchiolitis obliterans syndrome. (**b**) Patients excluding those with bronchiolitis obliterans/bronchiolitis obliterans syndrome.

Obstructive LFI after HSCT developed in 24 patients. The cumulative incidence of obstructive LFI was 7.1% (95% CI: 3.8–11.9%) at 2 years, 15.4% (95% CI: 7.8–18.3%) at 5 years, 15.7% (95% CI: 10.3–22.1%) at 10 years, and 19.6% (95% CI: 11.1–29.8%) at 15 years (Fig. 2a). The median time to onset of obstructive LFI was 2.5 years (range: 0.2–14.6 years). During the study period, LFI resolved in only two patients. After excluding patients with BO/BOS, 13 patients developed obstructive LFI after HSCT. Of these, two symptomatic patients received inhaled drugs for chronic obstructive pulmonary disease (COPD). The cumulative incidence of obstructive LFI without BO/BOS was 0% (95% CI: 0–0%) at 2 years, 3.0% (95% CI: 1.0–7.0%) at 5 years, 10.6% (95% CI: 5.7–17.2%) at 10 years, and 35.6% (95% CI: 19.2–52.5%) at 15 years (Fig. 2b). The median time to onset of obstructive LFI without BO/BOS was 2.9 years (range: 0.2–14.6 years). Multivariate analysis identified HLA compatibility (HR: 4.74; 95% CI: 1.41–15.93; *P* = 0.01) and TBI in conditioning (HR: 0.23; 95% CI: 0.07–0.71; *P* = 0.01) as significantly associated with obstructive LFI (Supplementary Table 1).

**Figure 3:**
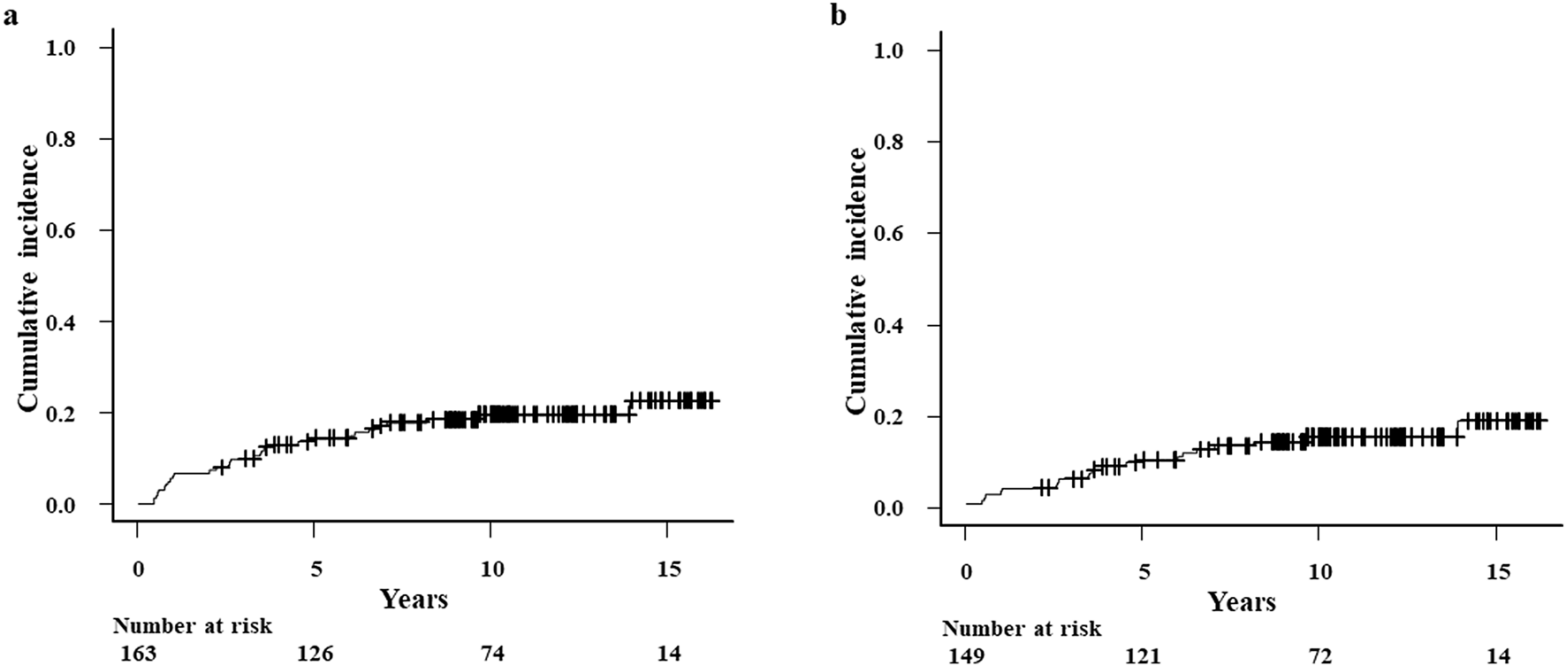
Cumulative incidence of restrictive lung function impairment. (**a**) Patients including those with interstitial disease/organizing pneumonia. (**b**) Patients excluding those with interstitial disease/organizing pneumonia.

Post-HSCT, 31 patients developed restrictive LFIs, which had a median time to onset of 2.6 years (range: 0.2–13.9 years) and a cumulative incidence of 7.4% (95% CI: 4.0–12.1%) at 2 years, 14.3% (95% CI: 9.4–20.2%) at 5 years, 19.5% (95% CI: 13.6–26.2%) at 10 years, and 22.7% (95% CI: 14.6–31.8%) at 15 years (Fig. 3a). Causal factors at the time of PFT were two of postoperative changes, one of non-tuberculosis mycobacterial lung disease, pericardial and pleural effusion due to serositis-type GVHD, lung cancer, and vertebral compression fracture. Six patients with restrictive LFI recovered. Of these, four (three with ILD and one with chronic GVHD) recovered after treatment. The remaining two patients recovered without any treatment. Ten and nine patients with restrictive LFI also had BO/BOS and ILD/OP, respectively. The cumulative incidence of restrictive LFI without ILD/OP was 3.4% (95% CI: 1.3–7.2%) at 2 years, 9.6% (95% CI: 5.5–15.1%) at 5 years, 14.5% (95% CI: 9.2–21.0%) at 10 years, and 18.0% (95% CI: 10.1–27.7%) at 15 years (Fig. 3b). The median time to onset of restrictive LFI without ILD/OP was 3.5 years (range: 0.2–13.9 years). Supplementary Table 2 shows that extensive chronic GVHD (HR: 3.46; 95% CI: 1.49–8.01; *P* = 0.004) and BO/BOS (HR: 16.32; 95% CI: 4.51–59.05; *P* < 0.001) were risk factors significantly associated with restrictive LFI in univariate analysis. In multivariate analysis, however, BO/BOS (HR 15.38, 95% CI: 4.35–54.46, *P* < 0.001) was the sole, independent risk factor for restrictive LFI.

### Overall survival, non-relapse mortality and cause of death

Survival analysis was performed for the remaining 152 patients after excluding patients with any LFI before HSCT. Significant risk factors against OS identified through univariate analysis were stem cell source; peripheral blood (vs. bone marrow) (HR: 2.78; 95% CI: 1.11–6.95; *P* = 0.03), extensive chronic GVHD (HR: 3.05; 95% CI: 1.29–7.24; *P* = 0.01), BO/BOS (HR: 5.65; 95% CI: 2.06–15.5; *P* < 0.001), ILD/OP (HR: 3.28; 95% CI: 1.10–9.78; *P* = 0.03), obstructive LFI (HR: 2.93; 95% CI: 1.13–7.58; *P* = 0.03), and restrictive LFI (HR: 8.68; 95% CI: 3.63–20.74; *P* < 0.001) (Table 3). On multivariate analysis, restrictive LFI was a significant risk factor for OS (HR: 7.11; 95% CI: 1.71–29.49; *P* = 0.007). Obstructive LFI (HR: 3.16; 95% CI: 1.13–8.79; *P* = 0.03) and restrictive LFI (HR: 11.81; 95% CI: 4.39–31.77; *P* < 0.001) were also significant risk factors for NRM in univariate analysis (Table 4). Multivariate analysis identified restrictive LFI (HR: 12.19; 95% CI: 2.34–63.46; *P* = 0.003) as a significant risk factor for NRM. We also performed survival analysis for patients excluding BO/BOS and ILD/OP (Supplementary Tables 3 and 4). Restrictive LFI was a significant risk factor for OS (HR: 5.61; 95% CI: 1.47–21.42; *P* = 0.01) and NRM (HR: 7.66; 95% CI: 2.08–28.20; *P* = 0.002).

**Table 3:** Characteristics of the patients and factors associated with overall survival.

Factors	Number	Univariate analysis			Multivariate analysis		
		HR	95% CI	*P* value	HR	95% CI	*P* value
**Age at transplantation**							
< 50 years	115	1			1		
≥ 50 years	37	1.47	0.56–3.84	0.44	3.65	0.96–13.79	0.057
**Sex**							
Female	62	1			1		
Male	90	1.36	0.55–3.37	0.51	1.22	0.44–3.33	0.70
**Refined disease risk index**							
Low/intermediate	126	1			1		
High/very high	26	0.79	0.23–2.69	0.71	0.46	0.11–2.00	0.30
**HCT-CI**							
< 3	140	1			1		
≥ 3	12	1.06	0.25–4.58	0.94	0.60	0.10–3.45	0.57
**HLA compatibility**							
Match	99	1			1		
Mismatch	53	0.89	0.36–2.20	0.79	1.53	0.45–5.22	0.50
**Donor type**							
Related	43	1			1		
Unrelated	109	0.48	0.20–1.13	0.09	0.61	0.10–3.88	0.60
**Stem cell source**							
Bone marrow	110	1			1		
Peripheral blood	26	2.78	1.11–6.95	0.03	1.61	0.29–9.11	0.59
Cord blood	16	0	0–Inf	1	0	0–Inf	1
**Conditioning regimen**							
Non-myeloablative	23	1			1		
Myeloablative	129	1.95	0.45–8.39	0.37	2.84	0.51–15.71	0.23
**TBI/TLI in conditioning**							
No	62	1			1		
Yes	90	1.66	0.64–7.28	0.30	1.28	0.39–4.21	0.69
**Acute GVHD (grade II–IV)**							
No	92	1			1		
Yes	60	0.88	0.36–2.11	0.77	0.58	0.19–1.77	0.34
**Chronic GVHD (extensive)**							
No	106	1			1		
Yes	46	3.05	1.29–7.24	0.01	1.71	0.61–4.80	0.31
**BO/BOS**							
No	142	1			1		
Yes	10	5.65	2.06–15.50	< 0.001	1.34	0.20–8.92	0.76
**ILD/OP**							
No	141	1			1		
Yes	11	3.28	1.10–9.78	0.03	0.82	0.15–4.39	0.81
**Obstructive LFI**							
No	130	1			1		
Yes	22	2.93	1.13–7.58	0.03	0.66	0.14–3.22	0.61
**Restrictive LFI**							
No	124	1			1		
Yes	28	8.68	3.63–20.74	< 0.001	7.11	1.71–29.49	0.007

*HR* hazard ratio, *CI* confidence interval, *HCT-CI* Hematopoietic Cell Transplantation Comorbidity Index, *HLA* human leukocyte antigen, *Inf* infinite, *TBI* total body irradiation, *GVHD* graft-versus-host disease, *BO* bronchiolitis obliterans, *BOS* bronchiolitis obliterans syndrome, *ILD* interstitial lung disease, *OP* organizing pneumonia, *LFI* lung function impairment.

**Table 4:** Characteristics of the patients and factors associated with non-relapse mortality.

Factors	Number	Univariate analysis			Multivariate analysis		
		HR	95% CI	*P* value	HR	95% CI	*P* value
**Age at transplantation**							
< 50 years	115	1			1		
≥ 50 years	37	1.07	0.34–3.41	0.90	2.78	0.41–18.72	0.29
**Sex**							
Female	62	1			1		
Male	90	1.69	0.60–4.70	0.32	1.64	0.47–5.78	0.44
**Refined disease risk index**							
Low/intermediate	126	1			1		
High/very high	26	1	0.29–3.46	1	0.56	0.08–3.70	0.54
**HCT-CI**							
< 3	140	1			1		
≥ 3	12	0.68	0.08–5.53	0.72	0.49	0.02–13.02	0.67
**HLA compatibility**							
Match	99	1			1		
Mismatch	53	1.01	0.38–2.70	0.98	1.50	0.42–5.36	0.54
**Donor type**							
Related	43	1			1		
Unrelated	109	0.50	0.19–1.30	0.16	1.83	0.30–11.32	0.52
**Stem cell source**							
Bone marrow	110	1			1		
Peripheral blood	26	3.21	1.17–8.78	0.02	3.50	0.55–22.08	0.18
Cord blood	16	0	0–0	< 0.001	0	0–0	< 0.001
**Conditioning regimen**							
Non-myeloablative	23	1			1		
Myeloablative	129	3.03	0.39–23.72	0.29	3.38	0.42–26.89	0.25
**TBI in conditioning**							
No	62	1			1		
Yes	90	1.63	0.57–4.65	0.36	1.12	0.32–3.95	0.86
**Acute GVHD (grade II–IV)**							
No	92	1			1		
Yes	60	0.61	0.22–1.70	0.34	0.39	0.09–1.57	0.18
**Chronic GVHD (extensive)**							
No	106	1			1		
Yes	46	2.11	0.84–5.31	0.11	1.02	0.33–3.14	0.97
**BO/BOS**							
No	142	1			1		
Yes	10	6.08	2.15–17.19	< 0.001	1.48	0.15–14.43	0.74
**ILD/OP**							
No	141	1			1		
Yes	11	4.39	1.31–14.76	0.02	0.96	0.13–7.28	0.97
**Obstructive LFI**							
No	130	1			1		
Yes	22	3.16	1.13–8.79	0.03	0.50	0.04–6.44	0.59
**Restrictive LFI**							
No	124	1			1		
Yes	28	11.81	4.39–31.77	< 0.001	12.19	2.34–63.46	0.003

*HR* hazard ratio, *CI* confidence interval, *HCT-CI* Hematopoietic Cell Transplantation Comorbidity Index, *HLA* human leukocyte antigen, *Inf* infinite, *TBI* total body irradiation, *GVHD* graft-versus-host disease, *BO* bronchiolitis obliterans, *BOS* bronchiolitis obliterans syndrome, *ILD* interstitial lung disease, *OP* organizing pneumonia, *LFI* lung function impairment.

Causes of death in this study were a secondary malignancy (n = 6), BO (n = 2), heart failure (n = 2), underlying disease (n = 1), chronic lung GVHD (n = 1), renal failure (n = 1), infectious pneumonia (n = 1), interstitial pneumonia (n = 1), acute pancreatitis (n = 1), multiple sclerosis (n = 1), infectious peritonitis (n = 1), car accidents (n = 1), sudden death (n = 1), and suicide (n = 1). Among these patients, 12 (57.1%) developed restrictive LFI post-HSCT. The median interval from the development of restrictive LFI to death was 3.5 years (range: 0.1–9.2 years). Patients with restrictive LFI were less likely to die from a second malignancy than patients without restrictive LFI (8.3% vs. 44.4%).

## Discussion

This study confirmed that post-HSCT pulmonary function declined over time, consistent with findings from studies on younger HSCT recipients^14,16^. As pulmonary function declines post-HSCT, the proportion of patients who develop obstructive and restrictive LFI increases, resulting in a cumulative incidence of 19.6% and 22.7% at 15 years, respectively. Generally, the incidence of LONIPC is thought to be higher in peripheral blood stem cell transplantation^12^. Although the majority of this study included bone marrow transplantation, not a few patients developed LFI. Especially, the cumulative incidence of obstructive LFI without BO/BOS showed steep increase from 10 to 15 years after HSCT. Since most LONIPC occur within 2 years after HSCT^12,13^, this finding suggests that LFI, at least in part, is another entity and long-term continuous PFT is warranted.

Because advanced BO/BOS is usually irreversible and is associated with a high mortality rate, early detection of high-risk patients may allow enhanced monitoring and pre-emptive or prompt therapy before significant lung dysfunction occurs. As up to 20% of patients with BO/BOS remain asymptomatic at diagnosis^34^, PFT is a very useful screening tool for early diagnosis^10,11^. Accordingly, the efficacy of PFT has been studied and proven mainly in the early after HSCT. However, our long-term follow-up study did reveal that some patients developed irreversible airway obstruction after HSCT regardless of BO/BOS. Clinically, they are usually considered to develop COPD; however, only three patients had 20 or higher pack-years, and six patients were never smokers. In the present study, HLA mismatch rather than smoking history, were significant predictive factors of obstructive LFI after HSCT. COPD is a systemic disease, and lung injury may be a part of a global, vascular process that damages other organs^35,36^. Martinez et al. suggested that the hematopoietic and immune systems are crucial to COPD progression^37^. Thus, we speculate that HSCT, along with miscellaneous complications, induces lung injury and airway obstruction which may, at least in part, be exacerbated by immune reactions.

Only BO/BOS was found to be an independent risk factor for restrictive LFI. Although a restrictive spirometric pattern can be caused by increased residual volume, in this study it was observed in only three patients, including one patient with OP and, therefore, cannot have a causal role in all cases of restrictive LFI. Moreover, it is possible that a mix of restrictive and obstructive lung processes may occur in BO/BOS^38^. Restrictive lung impairment is caused by multiple factors, including intrinsic (caused by lung parenchymal disorders) and/or extrinsic (caused by extraparenchymal disorders) factors. Although the pathogenesis of restrictive LFIs in patients with HSCT have been thought to center on ILD/OP^39^, they are more heterogeneous. This may account for the fact that LFI more frequently resolved in patients with restrictive rather than obstructive LFI. Causal factors can be readily detected if there is a single, apparent factor such as postoperative change, pleural effusion, and fracture. However, statistical analysis cannot always include all factors. If multiple, complex factors are present, many patients are needed for accurate statistical analysis. Indeed, factors that are causally linked to a spirometric restriction pattern often remain indeterminate owing to their complexity^8,12,24^. Palmar et al. also studied restrictive ling disease; however, their data are from patients with cGVHD, and their factors and prognostic impact have not been fully analyzed^40^. Future studies with a larger patient cohort are necessary to more accurately characterize the relationship between restrictive LFI and its causal factors.

Patients with restrictive LFI are at an increased risk of mortality from various causes, most commonly those other than an underlying disease or secondary malignancy. Of note, LONIPC was not the main cause of death. In this study, patients who developed LONIPC and died within 2 years were excluded. This finding suggests that the unfavorable prognostic impact of restrictive LFI cannot be explained by LONIPC only. We were unable to determine the reason for this, however, numerous population-based studies have reported that a restrictive spirometric pattern is associated with morbidity such as poor physical performance and cognitive impairment^41,42^, as well as all-cause mortality^23,43,44^. This high mortality rate may be caused not only by respiratory, but also by a systemic dysfunction. Pleural or pericardial effusion, which is caused by organ failure or severe serositis-type GVHD, could display a restrictive PFT pattern. Anorexia or steroid use also cause restrictive PFT pattern through respiratory muscle weakness in the long run. One large retrospective study of HSCT recipients (n = 2545) revealed that the presence of pre-transplant pulmonary restriction was significantly associated with NRM^8^. The authors speculated that this was partially caused by respiratory muscle weakness. Generally, frailty, including systemic muscle weakness seems to worsen after HSCT^45^. Therefore, restrictive LFI may not be the direct cause of death, but may reflect organ dysfunction or frailty.

Owing to its retrospective nature, our study has some limitations. First, although most patients who visited LTFU routinely received a PFT, several patients did not especially if they appeared to be healthy or dropped out of the LTFU. In addition, the number of PFT was decreased in 2020–2021 because of the coronavirus disease 2019 pandemic. These could affect the LME model analysis. Second, there was paucity of data on lung function after bronchodilator use. Third, our study included patients who survived for 2 years without relapse. In addition, our patient population was up to 2012 to ensure a long observational period. Thus, haplo-identical transplantation was not included, and mainly cases with bone marrow transplantation were assessed. Although this was favorable for investigating the transition of pulmonary function, it may have affected the survival analysis. Finally, LONIPC and LFI sometimes overlap each other and could complicate interpreting the analysis. As the sample size of this study is small, we could not perform multivariate analysis for patients without LONIPC to confirm the results. However, LFI and LONIPC often do not occur at the same time. In addition, they need LTFU periods to determine whether these overlap or not. Therefore, the multivariate analysis considering these events as time-dependent covariates together could be in line with the clinical setting. Owing to these limitations, our conclusion needs validation in a larger and prospective cohort.

In conclusion, this study confirmed that pulmonary function declined over time after HSCT and highlighted the clinical impact of newly developed LFI, regardless of the presence of LONIPC. Further, large-scale studies are warranted for establishing an appropriate treatment strategy.

## Supplementary Information

Supplementary Table 1.

Supplementary Table 2.

Supplementary Table 3.

Supplementary Table 4.

## Data Availability

The datasets used and/or analysed during the current study available from the corresponding author on reasonable request.
